# Constrained Fourier estimation of short-term time-series gene expression data reduces noise and improves clustering and gene regulatory network predictions

**DOI:** 10.1186/s12859-022-04839-z

**Published:** 2022-08-09

**Authors:** Nadav Bar, Bahareh Nikparvar, Naresh Doni Jayavelu, Fabienne Krystin Roessler

**Affiliations:** 1 Department of Chemical Engineering, Norwegian University of Science and Technology (NTNU), Sem Sælandsvei 4, Trondheim, NO-7491 Norway; 2 Division of Medical Genetics, Department of Medicine, University of Washington Seattle, Seattle, WA 98195-7720 USA

**Keywords:** Gene expression data, Fourier transform, Noise, k-means Clustering, Network component analysis, Time-Series data

## Abstract

**Background:**

Biological data suffers from noise that is inherent in the measurements. This is particularly true for time-series gene expression measurements. Nevertheless, in order to to explore cellular dynamics, scientists employ such noisy measurements in predictive and clustering tools. However, noisy data can not only obscure the genes temporal patterns, but applying predictive and clustering tools on noisy data may yield inconsistent, and potentially incorrect, results.

**Results:**

To reduce the noise of short-term (< 48 h) time-series expression data, we relied on the three basic temporal patterns of gene expression: waves, impulses and sustained responses. We constrained the estimation of the true signals to these patterns by estimating the parameters of first and second-order Fourier functions and using the nonlinear least-squares trust-region optimization technique. Our approach lowered the noise in at least 85% of synthetic time-series expression data, significantly more than the spline method ($p<10^{-6}$). When the data contained a higher signal-to-noise ratio, our method allowed downstream network component analyses to calculate consistent and accurate predictions, particularly when the noise variance was high. Conversely, these tools led to erroneous results from untreated noisy data. Our results suggest that at least 5–7 time points are required to efficiently de-noise logarithmic scaled time-series expression data. Investing in sampling additional time points provides little benefit to clustering and prediction accuracy.

**Conclusions:**

Our constrained Fourier de-noising method helps to cluster noisy gene expression and interpret dynamic gene networks more accurately. The benefit of noise reduction is large and can constitute the difference between a successful application and a failing one.

## Introduction

Any biological data we collect is corrupted to some extent by noise. Most scientists address this by using a variety of methods, all of which aim to reduce the noise in the signal and to increase the useful information stored in it. In molecular biology, reducing the noise of gene expression data requires the removal of some undesired elements that degrade the useful information stored in the measurements.

Time-series expression data has become important to the study of cellular network responses because the data contains both the gene expression levels and timings [1]. This data is also used with various techniques, such as gene clustering, principal component analysis (PCA), and network component analysis (NCA), all of which facilitate network decryption by analyzing the temporal gene expression patterns. But time-series expression data also contains noise, as each successive sample is subjected to variations in the cell culture/tissue, to genetic diversity, to different phases and amplitudes in the intracellular processes we are trying to study, and varying regulatory networks [1–3]. The problem of noise intensifies when we attempt to analyze time-series expression data. Due to restricted resources, we usually acquire only a limited number of time-variant samples. With few samples the noise can partially, or even completely, obscure the real signals. Thus, we run the risk of inferring wrong network dynamics from the fusion of noise and data. Nevertheless, noisy time-series data is often employed in clustering (e.g., k-means) and gene regulatory network analysis tools (e.g. NCA), without any form of a priori filtering, although these tools are noise-sensitive [4–7].

Several authors in the past decade proposed solutions to reduce the noise of time-series data. References [8–10] first assign genes to different classes (using either previous knowledge or clustering methods), then use cubic splines to model time-series data of one class with allowance for gene specific parameters. However, this method does not incorporate information about the temporal shape of the gene, and needs to estimate at least 5 parameters [11]. Huang and Sanguinetti [12] proposed the DICEseq, that explicitly models the correlations between different RNA-seq experiments, and transfers information between samples through a Gaussian process. It can enable an effective trade-off between sequencing depth (to improve the accuracy of each sample) and time points. Sloutsky et al. [4] relies on multiple measurement replicates, an expensive and resource-consuming procedure. They also proposed to use array data from similar cell lines (but different experiments) for clustering purposes, but this procedure introduces noise originating from other sources, such as genetic variation and different initial conditions. Zeisel et al. [13] proposed to reduce the number of replicates by introducing a noise model that detects differentially expressed genes (DEGs). Others suggested to incorporate robust noise models to array experiments [13, 14], but these models require normally distributed, independent noise among samples, conditions that are suitable for individual measurements but not always for time-series data. Many researchers attempted to reduce the noise for clustering purposes [7, 11, 15–17]. For instance, several authors explored Fourier expansion models combined with autocorrelation variance structures to increase the accuracy of gene clustering during the cell cycle [7, 16, 17]. The authors assume known Fourier periods in the data [17] that are generally obscured. We previously showed [18] that unconstrained Fourier approximation can improve post-processing applications, but that model could also produce inadequate frequencies that caused overfitting.

More recently, different methods to reconstruct the original shape of the genes were presented. For instance, [19] developed a model for time-series data using linear mixed model splines. They also developed a corresponding R package *lmms* that can be used for both microarray and RNA-seq gene expression data. Another bioinformatic group [20] developed a statistical model for clustering time-series data, a model which combines a Dirichlet process model with a Gaussian process model (DPGP). More specifically, the Dirichlet process incorporates cluster number uncertainty, whereas the Gaussian process models time-series dependencies. The authors show that the DPGP algorithm could successfully cluster noisy RNA-seq and microarray gene expression data. Other authors [21, 22] used an impulse model to describe time-series gene expression data. More specifically, the R package *ImpulseDE* [21] can be used for any type of high-throughput gene expression data while *ImpulseDE2* [22] was tailored for count data. A recent review paper [23] compares the performance of several recent algorithms including *lmms* and *ImpulseDE2* using synthetic and real RNA-seq data. In their comparison, *ImpulseDE2* was overall the best performing tool.

The short-term temporal pattern of gene expression over a time scale of several hours appears to follow a few basic shapes, which we can exploit to reduce its noise [24, 25]: (1) short impulses represent genes that are up- or down-regulated for short time periods, (2) sustained responses yield a change in the transcript level of a gene for a long period, and (3) basic wave patterns of one or two peaks. Because we can accurately approximate any wave or impulse-like shape by Fourier series, we proposed a method that constrains the fit of each gene to a temporal pattern that belongs to one or a combination of these two basic patterns. We approximated the temporal data using an optimal least squares trust-region method, a known optimization algorithm, and restricted the optimality search to frequencies that can construct these basic patterns. By doing so, we eliminated some of the noise in the data. We modeled noise in microarray data by a Gaussian model [13] and RNA sequencing (RNA-seq) data by negative binomial distribution [26, 27] (see In “Methods” section). We evaluated our algorithm using synthetically generated data with varying noise levels and showed that constrained Fourier approximation with single and second harmonics reconstructed 95% of the true signals accurately. We showed that downstream processing of our de-noised data becomes significantly more effective, including clustering and network analysis. Lastly, we demonstrated the efficiency of the noise reduction (NR) method on independent real datasets, each with two independent replicates. We showed that the downstream processing of our de-noised data with NCA yielded well correlated duplicates and produced results that are in accordance with current knowledge, in contrast to datasets with no noise treatment. Taken together, noise in time-series gene expression data must be reduced in order to exploit the full potential of genetic network analytical tools. We propose our constrained Fourier fit as a viable method to reduce the noise in gene expression data.

## Methods

### Assumptions on the temporal patterns of genes

It was shown in several previous works that there are clear patterns of gene expression, both in response-to-stimulus experiments, developmental studies and cell cycle experiments. Bar-Joseph et al. [1] show at least five families of clear temporal patterns during several hours. This was later reinforced by the work of Yosef and Regev [25], showing similar temporal dynamics in gene expression, both in eukaryotes and prokaryotes. We therefore assume that short-term temporal patterns of gene expression follow a few basic shapes over a time scale of several hours (5–48 h) [9, 24, 25]: (1) short impulses represent genes that are up- or down-regulated for short periods, (2) sustained responses yield a change in the transcript level of a gene for a long period, and (3) basic wave patterns of one or two periods. Fourier transform with constraints on the frequency can approximate these patterns with high fidelity. This assumption is based on the fact that genes with cyclic behavior rarely have more than two periods during a short time span (up to 48 h [28]).

### Assumptions on the noise

We assume that the noise of time-series gene expression data arises from [2, 29, 30] (1) the variability in biological samples taken from different tissues (in case of mammalian cells) or cultures (bacteria and yeasts), (2) the variability of cells in each tissue, (3) variations in processes and genes, such as varying phases and amplitude, different responses to stimuli, feedback loops and networks, (4) that cells are not always arrested at the same time, (5) the handling of measurements, in which the samples are not obtained identically, and (6) some genes display a delay of more than 20 min between completing transcription and mRNA production [29]. Microarrays were particularly prone to measurement noise [13], but the RNA-seq techniques also suffer from noise inherited in biological samples [29]. The noise in microarray data was previously shown to be normally distributed (by Kolmogorov–Smirnov test) with both additive and intensity-dependent terms [13]. We therefore tested both proportional and additive random noise models in the following manner:

1 $$\begin{aligned} y_z=v_z[1+c_1(z)]+c_2(z) \end{aligned}$$

where $y_z$ are the measurements, $z=1,\ldots ,m$ , $v_z$ are the corresponding real signals, $c_1(z)$ and $c_2(z)$ are normally distributed noise values with variance $\sigma ^2$ and $\phi ^2$, respectively. This noise model accounts for noise that is proportional to the signal strength and an additive term, but does not account for phase shifts that may be present due to asynchronous cells, mostly because we do not have information on phase shift in time-series experiments. We also tested additive noise (i.e. $c_1(z)=0$).

It was shown, that the variance of noisy RNA-seq data increases with the gene expression in a negative binomial distribution manner [26, 27]. We implemented this variance by approximating a negative binomial distribution for the RNAseq data with the following function:

2 $$\begin{aligned} \sigma ^2_z=\rho + \kappa _1 v_z^{\kappa _2} \end{aligned}$$

with $v_z$ being the normalized real gene expression levels, $\kappa _1$, $\kappa _2$ and $\rho$ were estimated using the Matlab Fitting Toolbox from a negative binomial distribution [31]. Normalization was conducted by dividing at each time sample, all the gene expression replicates by the library size at that time sample.

### Fourier estimation with nonlinear least squares trust-region

To fit the data points of each gene in an optimal manner, we use nonlinear least squares to estimate the parameters $a_0$, $a_i$ and $b_i$ for $i=1,2,\ldots n$ of a Fourier function of degree *n*,

3 $$\begin{aligned} h(x,t)=a_0 + \sum _{i=1}^{n} a_i \cos (2\pi it/\omega )+b_i \sin (2\pi it/\omega ) \end{aligned}$$

where $\omega$ is the period of the signal, $a_0$ is the constant term of the data and is associated with the $i = 0$ cosine term, and $1 \le n \le \infty$ is the number of harmonics (order) in the series. *x* is the vector of parameters $\omega , a_i,\ b_i$ for $i=0,1,\ldots ,n$. Our objective is therefore to find the Fourier curve that minimizes the error between our data and the curve for each set of time-series expression. We stress that higher orders ($n\ge 3$) require estimation of at least eight parameters (compared with five in spline methods) and may yield over-fitting for low number of samples (see In “ Discussion” section).

Let the observed expression values of the gene at time points $t_1,\ldots ,t_m$ be $y_1,\ldots ,y_m$, where *y* has the form

$$\begin{aligned} y=h(x,t) \end{aligned}$$

where $x\in {\mathbb {R}}^n$ is the vector of parameters. We need to find the optimal parameter vector $x^*$ such that *h* best fits the data in the least squares sense

4 $$\begin{aligned} f(x)=\frac{1}{2} \sum _{z=1}^{m} [ h(x,t_z)-y_z ] ^2 =\frac{1}{2} r(x)^T r(x) \end{aligned}$$

where *r*(*x*) are the residuals, so that $x^*$ is found by minimizing *f*(*x*).

In reality, our time-series expression data contains measurement error. We account for that by redefining *f*(*x*)

5 $$\begin{aligned} f(x,\tau )=\frac{1}{2} \sum _{z=1}^{m} [(h(x,\tau _z)-y_z)^2+(\tau _z-t_z)^2 ] =\frac{1}{2} [ r(x,\tau )^T r(x,\tau )+e(\tau )^T e(\tau )] \end{aligned}$$

where $\tau =(\tau _1,\ldots ,\tau _m)$, and where $r(x,\tau )$, and $e(\tau )$ are the *m*-vectors composed of $r_z(x,\tau )=h(x,\tau _z)-y_z$ and $e_z(\tau )=\tau _z-t_z$, respectively. Here, we repeat the known trust-region method from optimization theory [32]. The gradient and the Hessian of *f*(*x*) are

6 $$\begin{aligned} g(x)&= \nabla f(x)=A(x)r(x) \end{aligned}$$

7 $$\begin{aligned} H(x)&= \nabla ^2f(x) \end{aligned}$$

8 $$\begin{aligned}&= A(x)A(x)^T+\sum _{z=1}^{m} r_z(x) \nabla ^2 r_z(x) \end{aligned}$$

The idea is to adjust the Fourier coefficients in *x* such that *f*(*x*) decreases for each iteration. At the iteration *k*, the step $\delta ^{(x)}$ is the solution to the system

9 $$\begin{aligned} (A_kA_k^T+\mu _kI)\delta =-g_k \end{aligned}$$

for some $$\mu _k\ge 0$$.

Let $\delta ^{(k)}$ be the solution of the system. Then $\delta ^{(k)}$ solves the trust-region subproblem [32]:

10 $$\begin{aligned} \min \quad q_k(\delta )&= f_k + g_k^T\delta +\frac{1}{2}\delta ^TB_k \delta \end{aligned}$$

11 $$\begin{aligned} \text {s.t.} \qquad \Vert \delta \Vert\le & \Delta _k\end{aligned}$$

12 $$\begin{aligned} \omega ^L_{i}&\le \omega _i \le&\omega ^u_{i} \end{aligned}$$

with $\Delta _k=\Vert \delta ^{(k)}\Vert$, $\omega ^L_{i}$ and $\omega ^u_{i}$ are the lower and upper limit, respectively, on the Fourier frequencies. In the case of only one Fourier harmonic, $i=1$. The solution of $\delta ^{(x)}$ can be obtained by controlling the radius $\Delta _k$ but the choice of this radius is not trivial. It should be large enough so convergence will be reasonably fast, but ensure that $q_k(\delta )$ adequately approximates *f*(*x*). For this, we compare between the actual and predicted reduction by the following manner [32]:

13 $$\begin{aligned} ared(\delta ^{(k)})=f(x^{(k)})-f(x^{(k)}+\delta ^{(k)}) \end{aligned}$$

and the quadratic model

14 $$\begin{aligned} pred(\delta ^{(k)})=f(x^{(k)})-q_k(\delta ^{(k)}) = -g_{k}^{T}\delta {^{(k)}}-\frac{1}{2}\delta {^{(k)T}} H_k \delta ^{(k)} \end{aligned}$$

If the step $\delta ^{(k)}$ is not acceptable, then we reduce $\Delta ^{(k)}$ to improve the accuracy and re-compute the step $\delta ^{(k)}$. The optimal nonlinear least squares approximation method that fits the curve to the time-series expression data can be summarized by the algorithm in the next section.

### The algorithm

For every gene in the dataset, repeat the following conceptual algorithm:
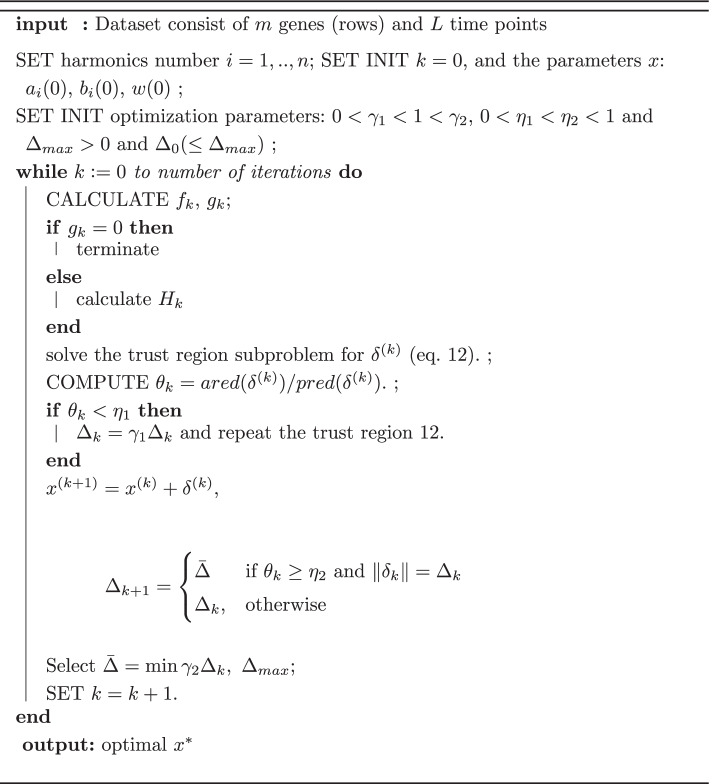


Several other improvements and strategies of the trust region are discussed in [32]. Unlike previous unconstrained Fourier approximations methods [18], and to comply with the assumptions on the temporal patterns of gene expression [9], we assume that the fitted function will consist of no more than two amplitudes (corresponding to wave) and at least 1/4 period (corresponding to sustained response). We therefore constrained the search of $\omega _i$ in the iterative nonlinear least squares to the interval $[ 0.25\pi /t_e,\ 4\pi /t_e ]$, where $t_e$ is the time span of the experiments (hours). We used the optimization and curve fitting toolboxes in Matlab (Mathworks Inc., Massachusetts, U.S.A).

### Synthetic data for comparison with other de-noising methods

To evaluate our algorithm, we first compared the performance of the constrained Fourier estimation, to the performance of the common spline smoothing method on synthetic microarray data (normally distributed noise). For that, we analyzed the performance of algorithm 1–100 increasingly frequency ($\omega$) values in Eqs. 3 and 12. For synthetic RNA-seq data (negative binomially distributed noise), we compared the performance of the constrained Fourier estimation to the R package *ImpulseDE* [21], another tool that was developed for both microarray and count data. *ImpulseDE* first groups genes into a limited number of clusters. Afterwards, an impulse model is fit to the mean expression profile of each cluster which is then used as a starting point for fitting the impulse model to each single gene separately.

For the comparison, we calculated the discrepancy between the curves (Fourier vs. spline estimations and Fourier vs. *ImpulseDE*) by the sum of squared error (SSE) and the root mean squared error (RMSE). Our constrained Fourier estimation does not rely on SSE, it restricts the signals to certain frequencies and exits by the condition in Eqs. 13–14. Because the true signals were known in the synthetic cases, we used the SSE for comparison.

Each experimental replicate provided a measurement matrix *E* of gene expression. We assume that replicates of real noisy data should produce similar principal components, must be clustered similarly, and should result in similar regulation patterns. For a similarity of noisy genes (true signal is unknown) and the evaluation of post-processing, we used (1) correlation coefficient to evaluate whether genes can be grouped together [11], since the correlation consider the shape and phase of the genes, and not the amplitude. This measure is important because network component analysis is only accurate up to a scaling factor [33]. (2) Angle between vector subspaces provide a quantitative measure for asserting genome-wide similarities. Orthogonal angles when similarity is expected or claimed raise questions about the validity of the hypothesis under examination [34]. Generally, the angle between the subspaces of two experimental predictions provides a measure of the amount of new information that is introduced by the second experiment not associated with statistical errors of fluctuations [34]. Although obtained differently, the Pearson correlation and the angle between subspaces are closely related.

### Synthetic data for downstream data processing

We created synthetic data to evaluate the performance of post-processing such as clustering and network component analysis. In order to evaluate clustering performance, we first generated 6 non-correlated ($p<0.2$) signals with random frequencies between $0.1\pi$ and $4\pi$. We then applied the random noise of Eq. 1 to each signal (see Additional file 1: Fig. S2). It was shown that experimental design is important and the sampling frequency has large implication on the true signal discovery [12]. To test the efficiency of noise reduction with increasing sampling frequency, we sampled the signals (1) linearly between 0 and 24 h and, (2) with a logarithmic scaled frequency, taken at 0, 1 min, 5 min, 10 min, 20 min, 40 min, 1 h, 2 h, 4 h, 8 h, 12 h, 16 h, and 24 h. These sampling distributions are consistent with microarray and RNA-seq time-series experiments [28, 35, 36].

To measure the performance of the NCA, we first generated 1000 synthetic signals with random frequencies (between $0.1\pi$ and $4\pi$, uniformly distributed) and with random amplitude (normally distributed with mean zero and variance 0.5). We sampled these as described above. For each gene, we generated three replicas by introducing additive Gaussian noise. We also generated 100 random network topology matrices $A_0$ (with additive Gaussian noise). We then evaluated the ability of NCA to reconstruct the activity of the regulators *P* from the three *E* replicates by

15 $$\begin{aligned} E=AP + \Gamma \end{aligned}$$

where $\Gamma$ is the process noise. The full method is described in [33, 37, 38]. We hypothesized that for any identical network topologies (matrix *A*), reconstruction of the regulators (matrix $P \ \in {\mathbf {R}}^{m\times l}$, with *m* number of regulators and *l* number of time samples) should be identical up to a scaling factor (see [33]) for all the replicates. We used Pearson correlation for similarity measure.

To compare the performance of de-noising (smoothing) methods that first employ clustering [2] to methods that estimate individual genes using Fourier optimization we presented, we used k-means++ clustering algorithm by matlab (Mathworks Inc.). It uses a two-phase iterative heuristic algorithm to find centroid seeds and to minimize the sum of the point-to-centroid distances. We randomly generated six clusters with low correlation ($p<0.4$), and created at least 100 random signals for each cluster by adding white noise (mean zero, $\sigma ^2>0.1$). We then compared the performance of two groups: (1) cluster the 600 signals to 6 groups with k-means, followed by denoising according to each cluster’s mean (Additional file 1: Fig. S1, black arrows), with (2) de-noise individual genes with algorithm 1, and then cluster the treated signals with k-means into 6 groups (Additional file 1: Fig. S1, blue arrows). We evaluated the performance of the two approaches by (1) the discrepancy of mean shape at each cluster with the original cluster (see *SSE* below), and (2) mean correlation of the approximated signal to the true signal.

The k-means++ is a stochastic algorithm since it selects *k* *random* initial cluster centroid positions from all the signals. We therefore run Monte Carlo simulations ($n=6000$) and compared the error (SSE) of the raw data with the de-noised data by the following

16 $$\begin{aligned} SSE=\sqrt{\sum _{i=1}^{n} (S_i-m_i )^2 } \end{aligned}$$

with $S_i$ is the mean of all the noisy signals in a cluster *i*, and $m_i$ is the mean of the signals that were clustered by the k-means algorithm.

To calculate the accuracy of k-means clustering the real data, we used the silhouette measure, that indicates how similar a point is to points in its own cluster, when compared to points in other clusters. The silhouette value $s_i$ for the *i* point is defined as

17 $$\begin{aligned} s_i = \frac{b_i-a_i}{\max (a_i,b_i)} \end{aligned}$$

where $a_i$ is the average distance from the *i* point to the other points in the same cluster, and $b_i$ is the minimum average distance from the *i* point to points in a different cluster, minimized over clusters. The distance between each two points $x_j$ and $y_j$ is calculated by

18 $$\begin{aligned} d=\sum _{j=1}^{n} |x_j-y_j| \end{aligned}$$

where *n* is the number of points to consider. The values of the clustering accuracy and the centroid distances were averaged over the number of trials in our Monte Carlo simulations.

All the significance tests were conducted with Welch’s two-sample *t*-tests for the signals ($n>600$) that were generated from a normal distribution. We tested the alternative hypothesis that the population means are not equal.

### Testing the algorithm with real expression data

To test the algorithm with real expression data, we used a time-series RNA-seq data from the bacterium *Listeria monocytogenes* strain ScottA, induced with high pressure shock of 400 MPa during 8 min at 8 $^\circ$C. The data is available in the European Nucleotide Archive (ENA) under accession code PRJEB34771 [39]. The data provides the gene expression level for 2953 differentially expressed genes (DEGs) of the ScottA strain with at least three replicates for each untreated/treated sample at 9 time points (0, 5, 10, 30, 45 min, 1, 6, 24, and 48 h) post treatment.

Additionally, we used microarray data of mouse T cells treated with interleukin-2 (IL-2) at 10 time points over a period of 0–24 h was downloaded from GEO database with accession number: GSE6085 [35]. The data was processed using limma package in R/Bioconductor [40]. The expression values were averaged over replicate measurements. The DEGs were identified with fold change (FC) > 1.5 and adjusted *p* value < 0.05 at minimum two time points.

## Results

### Synthetic gene reconstruction

**Fig. 1:**
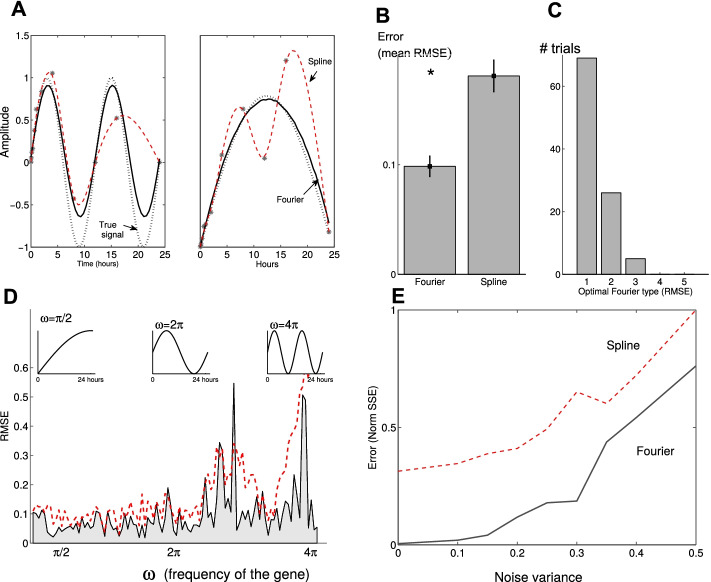
Constrained Fourier approximation fit the gene expression data accurately. **A** Two examples of true signals (dotted curve), noisy data (’*’), Fourier approximation (solid) and the spline approximation (red dashed) for frequencies of $4\pi$ (left) and $\pi$ (right). Spline approximations follow the noise. **B** The root mean squared error (RMSE) is significantly (two-samples *t*-test, $p<10^{-6}$, $n=100$) lower for the Fourier approximation than the spline. Furthermore, **C** 85% of the trials were accurately approximated (lowest RMSE) by Fourier with first and second harmonics. **D** Frequency analysis of the Fourier approximations: The error is low for frequencies $<3\pi$, but increases with frequency. The spline approximation (red) is higher, with its mean (mean RMSE of all frequencies) significantly ($p<10^{-5}$) higher than the Fourier. A sustained stimulus, an impulse and a wave-like response with frequencies $\pi /2$, $2\pi$ and $4\pi$, respectively, are depicted above. **E** Deterioration of the noise reduction methods (expressed by the normalized sum of SSE) as the noise variance $\sigma ^2$ of the gene expression measurements increases. Fourier algorithm performs better than its counterpart for all variances tested

To compare between the Fourier and the spline approximations of the true signal, we generated a sequence of 100 noisy signals with variance $\sigma ^2=\phi ^2=0.5$ and increasing frequency (see “Methods” section). Spline follows the noisy data and produces high correlation, but does not reconstruct the true signal adequately (see Fig. 1A for two selected signals). In contrast, the constrained Fourier approximation was significantly more accurate than spline (two sample *t*-test, $p<10^{-6}$), and managed to reconstruct the true signals accurately (RMSE $<0.1$) for 80% of the frequencies we tested (Fig. 1B). In fact, Fourier fit with only one harmonic was superior to the other Fourier harmonics and the spline methods (Fig. 1C) for the signals and noise we tested. It was shown previously [18] that Fourier approximation of the data by two harmonics ($n=2$) yielded good results. In their results, the fit was unconstrained, not taking into account the intrinsic shape of the data (see “Methods” section). In fact, when we compared the reconstruction error of Fourier fits with several harmonics, we found that the first and second approximated the data accurately (85% of the trials, Fig. 1C). In contrast, the fourth and fifth harmonics failed to reconstruct the noisy data completely (Fig. 1C).

Fourier approximations with one or two harmonics accurately reconstructed the signals at low frequencies (we tested up to $4\pi$, Fig. 1D), yielded low error (RMSE), but suffered from higher errors at frequencies higher than $3\pi$. The constrained Fourier approximation yielded a better fit than the spline for almost all frequencies (Fig. 1D). The error (measured as $\Vert \text {RMSE}\Vert _2$ of all frequencies, see “Methods” section) of the constrained Fourier was two fold lower than the spline.

We also studied and compared the performance of the two methods to increasing data noise (increased variance $\sigma ^2$ and $\phi ^2$, see “Methods” section). We found that the approximations of both Fourier and spline tend to deteriorate as the variance increases, but the constrained Fourier consistently reconstructed the signals more accurately (Fig. 1E).

We also compared our constrained Fourier noise reduction method to a recent algorithm *ImpulseDE* [21] using synthetic RNA-seq data. We found that both algorithms successfully created consistent clusters of genes from the noisy data (Additional file 1: Fig. S3). More importantly, as the noise level increased with the expression level, the constrained Fourier algorithm was significantly more consistent with better correlation to the true signal and lower SSE (see also Table 1 and Additional file 1: Table S1). Interestingly, for the double top impulse expression shape (cluster 4 in Additional file 1: Fig. S3), the Fourier algorithm displayed low SSE ($0.47\pm 0.06$) and high mean correlation ($\rho =0.96\pm 0.01$) whereas the *ImpulseDE* displayed poorer results (mean SSE = $1.08\pm 0.04$ and $\rho =0.65\pm 0.03$). This was particularly more pronounced for “high noise” time points (right columns of Table 1 and Additional file 1: Table S1) which demonstrates that the Fourier performs particularly well when the noise in the data is high. This is also apparent in the low standard deviation resulted from the constrained Fourier of the 1000 synthetic genes (see right columns of Table 1).

**Table 1:** Comparison of mean correlation coefficients $\rho$ between noisy gene profiles and de-noised gene profiles using ImulseDE or our constrained Fourier approximation

	All time points			“High noise” time points		
	Noisy data	ImpulseDE	Constrained Fourier	Noisy data	ImpulseDE	Constrained Fourier
Cluster 1	0.97	0.99	0.99	0.68	0.82	0.91
	$(\pm 0.02)$	$(\pm 0.02)$	$(\pm 0.01)$	$(\pm 0.22)$	$(\pm 0.22)$	$(\pm 0.18)$
Cluster 2	0.94	0.98	0.99	0.73	0.90	0.97
	$(\pm 0.04)$	$(\pm 0.02)$	$(\pm 0.02)$	$(\pm 0.17)$	$(\pm 0.13)$	$(\pm 0.08)$
Cluster 3	0.97	0.99	1.00	0.80	0.95	0.99
	$(\pm 0.03)$	$(\pm 0.01)$	$(\pm 0.00)$	$(\pm 0.16)$	$(\pm 0.05)$	$(\pm 0.01)$
Cluster 4	0.97	0.65	0.96	0.79	0.32	0.90
	$(\pm 0.03)$	$(\pm 0.03)$	$(\pm 0.01)$	$(\pm 0.19)$	$(\pm 0.13)$	$(\pm 0.05)$
Cluster 5	0.93	0.98	0.99	0.58	0.82	0.91
	$(\pm 0.04)$	$(\pm 0.02)$	$(\pm 0.01)$	$(\pm 0.19)$	$(\pm 0.12)$	$(\pm 0.08)$
Cluster 6	0.98	0.99	0.95	0.92	0.96	0.98
	$(\pm 0.02)$	$(\pm 0.01)$	$(\pm 0.00)$	$(\pm 0.10)$	$(\pm 0.05)$	$(\pm 0.03)$

Standard deviation is shown in brackets.

### Behavior of clustering and network prediction tools

We studied how our constrained Fourier method affects post processing of data. For this, we used random generated (synthetic) data to compare the performance of clustering and network component analysis (NCA, [33]) computed with noisy and treated data. These two post analysis tools are widely employed to study networks from expression data.

#### *k*-means clustering

**Fig. 2:**
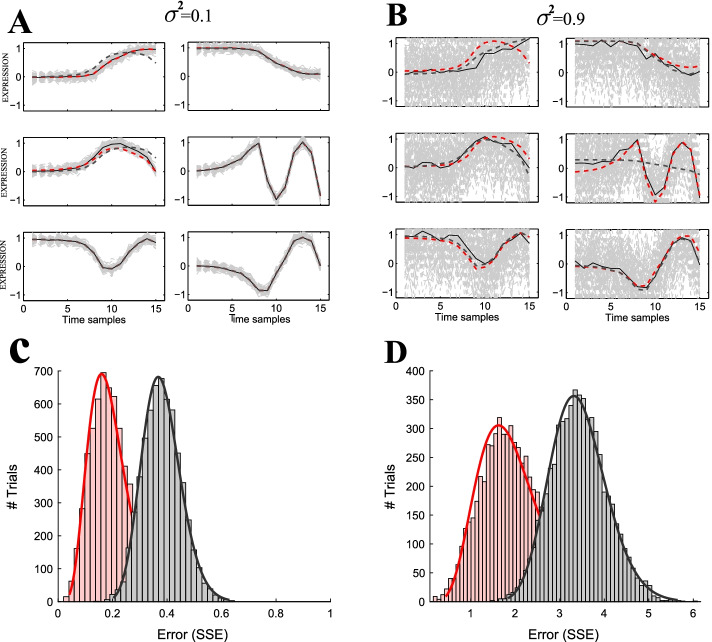
Results of k-means clustering of raw (gray) and de-noised (red) synthetic expression data. **A**, **B** Six synthetic clusters, from each we generated 1000 signals with random additive noise of variance $\sigma ^2=0.1$ (**A**) and $\sigma ^2=0.9$ (**B**). Fourier approximation of de-noised data that was clustered (red dashed) and Fourier approximation of raw data that was clustered (gray dashed). **C**, **D** Monte Carlo of 1000 k-means simulations (see “Methods” section) on the de-noised and raw signals. The histograms describe the distribution of the SSEs for the raw (grey) and the de-noised (red) data. The mean error SSE of Fourier treated genes (${\bar{SSE}}=1.9$) was significantly lower (*t*-test: $p<0.01$) than the mean SSE of the untreated genes (${\bar{SSE}}=3.4$).The difference in low noise signals (here shown $\sigma ^2=0.1$) was also statistically significant (*t*-test: $p<0.01$)

We tested and analyzed the accuracy of k-means clustering of raw data with de-noised data. The first analysis consisted of six selected, non-correlated ($r<0.2$) signals that are common in gene expression [1, 2, 9], i.e. sustainable response (Fig. 2A, B), impulse, double top impulse, inverse impulse and a wave. In the second analysis we randomly selected six composite signals (Fourier with one and two harmonics). In both analyses, we generated 100 signals for each of the clusters by adding white noise with variance $\sigma ^2$ (see “Methods” section), and tested the ability of k-means algorithm to re-construct the original six clusters from the noisy and de-noised data. We found several important observations.

Not surprisingly, at low variance the overall clustering performance (in terms of SSE and correlation, see “Methods” section) with the de-noised data was similar to clustering from raw signals (Fig. 2C, two sample *t*-test: $p<0.01$). More important however, reconstruction of the six clusters from higher variance signals ($\sigma ^2=\phi ^2=0.9$, Fig. 2D) showed that de-noised data performed better: The mean SSE for the de-noised data was significantly ($p<0.01$) lower than the mean SSE for the raw data. Correlation of the mean of the raw signals to the true clusters (depicted by solid lines in Fig. 2B) was particularly bad for the fourth cluster with two harmonics at higher variances. Additionally, large number of reconstructions (>100) from the raw-data resulted in a large SSE (>0.4), implying poor performance of k-clustering compared to the de-noised data.

**Fig. 3:**
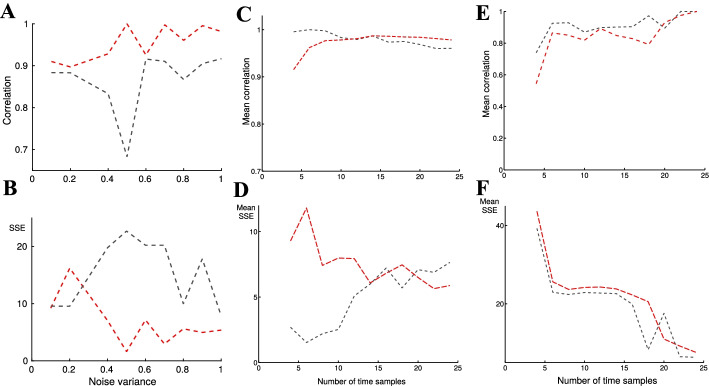
Analysis of k-means clustering of raw (gray) and de-noised (red) synthetic expression data. **A**, **B** Total size (of all six clusters) of correlation and SSE between the raw signals to the true signals (gray) and de-noised signals to the true signals (red). **C**–**F** analyzes the performance of the clustering as a function of the number of data samples: **C**, **D** Total mean correlation and error (SSE) of expression signals from clusters of raw (gray) and de-noised (red) data, as a function of sampling frequency (linearly distributed). The difference is not statistically significant for over 7 sample points (two-sample *t*-test). **E**, **F** Mean correlation and SSE of clustering of raw (gray) and de-noised (red) data, as function of sampling frequency with a logarithmic time scale (see “Methods” section). The improvement in the clustering performance was significantly better over 5–7 sample points. And most importantly, above 7–8 samples the improvement is not statistically significant

This analysis of the correlation and the SSE error was consistent when we gradually increased the variance (Fig. 3). Correlation of the de-noised signals were consistently high, even as the variance increased (Fig. 3A). Similarly, the SSE of the de-noised signals were stable and low as the variance increased (Fig. 3B), indicating robustness to noise variance.

Moreover, we found that the sampling frequency and distribution influenced the clustering performance. At low sampling (less than 7 time samples), k-means clustering of raw data outperformed (SSE and correlation) clustering Fourier de-noised genes at variance $\sigma ^2=0.5$. Over 8 samples, clustering Fourier approximated signals were indifferent (Fig. 3C, D). Moreover, when time samples were collected at a logarithmic scale, clustering performance was significantly improved for time-series with more than 5 samples (Fig. 3E, F).

#### Network component analysis

We tested two common NCA algorithms, the ROBNCA [41] and the GNCA-r [42]. Briefly, we generated three replicates of expression data using Gaussian noise, and tested the ability of NCA to reconstruct the regulator from the expression data in a consistent manner. We hypothesized that identical network topologies must reconstruct the signals identically for all replicates in the ideal case of noise filtration (see “Methods” section).

**Fig. 4:**
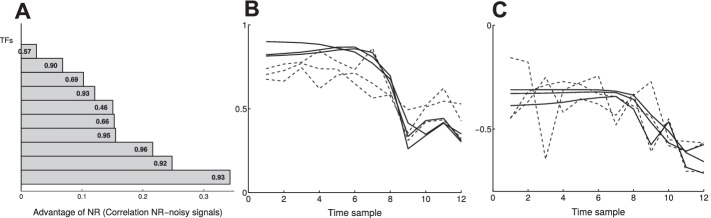
Post-processing with NCA performs better when data was treated with NR. **A** When reconstructed 10 transcription factor (TF) signals from 3 replicates of data, the correlation between the replicates was always higher when the data was first treated with our constrained Fourier estimation. Here we show noise variance $\sigma ^2=0.3$. Other variances and the GNCA-r are shown is Additional file 1: Fig. S5. Numbers besides column are the correlation of 3 replicates from treated data. **B**, **C** GNCA-r reconstructed the 3 replicates from the pre-treated (solid) data significantly better ($p<10^{-10}$) than the noisy data (dashed). Here we show temporal reconstruction of two arbitrary TFs

We found that the NCA algorithms consistently predicted similar TF signals from noisy replicates of data when the data was first treated by constrained Fourier (Fig. 4A–C). Not surprisingly, we found that noise treatment is increasingly important with increasing variance in the data (Additional file 1: Fig. S5), and our simulations strongly indicate that the NCA we tested cannot predict the TF temporal activities consistently when the data is noisy. For instance, NCA that predicted exact (Pearson $\approx 1$) temporal activity from three clean replicates (no noise), predicted unequal temporal activities (cross correlation elements $p<0.3$, Additional file 1: Fig. S5).

### Study of real biological time-series data

**Fig. 5:**
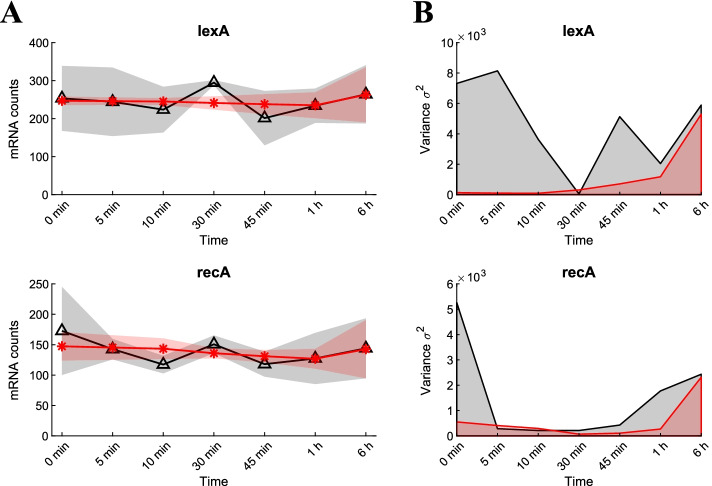
Noise reduction of Listeria monocytogenes RNA-sequencing differentially expressed data. **A** The variation in the mRNA counts between five replicates of the important early-active regulator genes lexA and recA was significantly reduced for the first 1 h after exposure to stimuli, reflected by the low variance **B** of the same genes at these early samples. Black triangles and red stars represent mean values for untreated and de-noised data, respectively. Shaded areas around mean values represent standard deviation

Firstly, we tested the ability of our algorithm to remove noise from bacterial gene expression data. The raw data provides a time-series mRNA counts of *Listeria monocytogenes* exposed to high pressure stress (400 MPa, 8 min, 8 $^\circ$C) [39]. Exposure to high pressure can induce SOS response, a global response to DNA damage to arrest the cell cycle until a full DNA recovery is accomplished [43, 44]. It has been shown that the induction of the SOS response is in the first phase of the bacterial response to high pressure which likely contribute to survival [45]. We selected two main regulators (transcription factors) of the SOS response, i.e. LexA and RecA with well known dynamics [46, 47], and investigated the impact of the de-noising algorithm on the expression data for the genes encoding for these two regulators. Figure 5A shows the average of mRNA counts (for 7 time points 0, 5, 10, 30, 45 min, 1, and 6 h after pressure treatment) for the genes *lexA* and *recA* before and after applying the de-noising algorithm with black triangles and red stars, respectively. The gray and red shaded area illustrate the standard deviation from the average count at each time point. According to the analysis, the de-noising algorithm could remove large noise associated with these early time points (0, 5, 10, 30, 45 min, and 1 h) such that the maximum standard deviation of the counts at these points decreased from 90 counts to no more than 15 counts by de-noising, both for the genes lexA and recA. The removal of noise especially in early time points was confirmed when comparing the variance of the untreated and the denoised data (Fig. 5B).

**Fig. 6:**
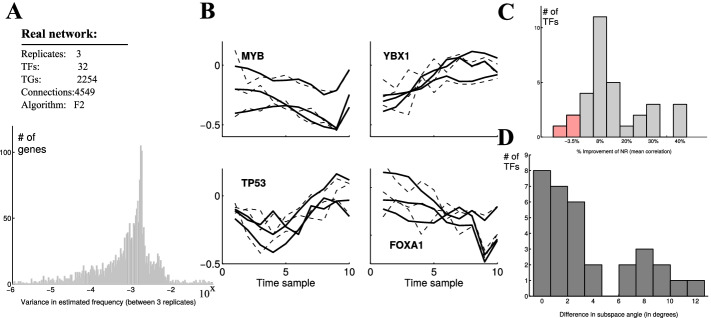
Post-analysis of mouse T cell expression data. **A** The algorithm estimated the data with 2-harmonics Fourier approximation. The mean variance of the estimated frequency $\omega$ between 3-replicates of each gene (log scale). 98% of the distribution had variance less than 0.01, indicating similar estimated frequencies between experiment replicates. **B** Selected TF activity predictions (using NCA) of noisy data (dashed) and Fourier de-noised data (solid). Replicates of Fourier estimated data are closely correlated (data on min and max cross correlation is given in Table 1). **C** Over 90% (29/32) of the TF activities had closer correlation (percent) with Fourier de-noised data than with noisy data. **D** Noisy data had exclusively higher mean angle between the replicates than the de-noised data, indicating that replicates of NCA predictions with de-noised data are more linearly dependent, and are closely related

Secondly, we evaluated the de-noising effect on 3 replicates of real mice T-cells time-series microarray data, and estimated the true signal with two-harmonics Fourier function (see “Methods” section). We found low variance between the frequencies of the three replicates (Fig. 6A): At least 98% (2095/2142) of the gene signals exhibited variance less than 0.01, with an average of 0.004 Hz. The NCA predicted similar TF activities from the three replicates data that were treated with the Fourier approximation (Fig. 6B–D). Comparison of four selected TF activities shows high cross correlation between the 3 replicates after de-noising (Table 2), in contrast to predictions with noisy data. In fact, less than 10% (3/32) of the TFs activity predictions exhibited worse correlations in the de-noised data, and even then, they performed no more than 3% worse (red columns, Fig. 6C). We also measured the similarity by the angle between the vectors, and found that the de-noised data yielded more similar predictions that the noise data, with lower mean angle between the replicates vectors (Fig. 6D).

**Table 2:** Cross correlation between three replicates of predictions of four TFs activities

TF	Treated data		Noisy data	
	Max	Min	Max	Min
MYB	0.86	0.48	0.68	0.29
YBX1	0.92	0.88	0.78	0.52
TP53	0.91	0.61	0.66	0.52
FOXA1	0.80	0.38	0.78	0.15

**Fig. 7:**
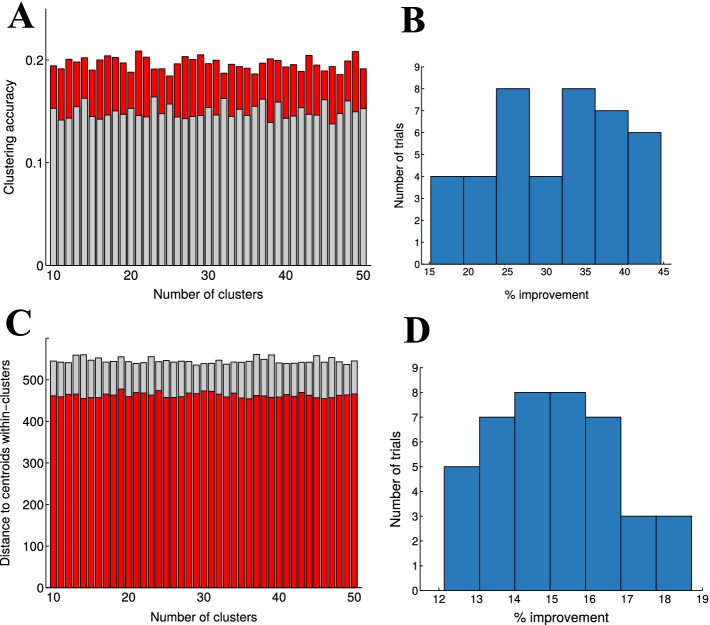
Post-analysis (k-means clustering) of mouse T cell expression data. **A** k-means clustering accuracy (Silhouette, see “Methods” section) of de-noised data (red) and raw data (gray) as a function of number of clusters tested from the real data. Difference was statistically significant ($p<10^{-10}$). **B** % improvement of k-mean clustering the de-noised data and the raw data. **C** The distance to centroids within clusters (calculated by within-cluster sums of point-to-centroid distances, see “Methods” section) of the de-noised data (red) and the raw data (gray) as a function the number of clusters. The de-noised data produced more centered clusters (results significantly different $p<10^{-10}$. **D** % improvement of the distance to centroids by using de-noised data

We evaluated the k-means clustering performance of the de-noised real data by testing increasing cluster numbers from 10 to 50. The k-means clustering of de-noised data produced more accurate clusters (in terms of silhouette, see “Methods” section) for each trial we tested, compared to clustering the raw expression data (Fig. 7A, B), with a significant ($p<10^{-10}$) improvement. The average distance to centroids within clusters (see “Methods” section) was significantly smaller (Fig. 7C, D) when clustered de-noised data, indicating more centered clusters.

## Discussion

Taken together, our results demonstrate an algorithm that eliminates some of the noise in time-series gene expression data. Our model exploits well documented common temporal gene expression patterns [1, 2, 9] to approximate the real signal shapes of individual genes with relatively high fidelity. The model relies on two key components: (1) The model solves nonlinear least squares optimization problem using trust-region method, that can account for constraints on the frequency and shape, and (2) the search for the original frequency is constrained by upper and lower bounds, i.e. $\pi /4$ and $\omega =4\pi$ during 24 h, an empirical evidence from large number of data [28]. These constrain the temporal shapes to sustained response, impulse shape and wave patterns.

Our results revealed several important issues: (1) Constrained Fourier accurately estimates cellular response to stimuli of the three temporal shapes we examined, and not only periodic (cyclic) signals as was suggested previously [7, 16, 17]. (2) Our method does not require knowledge of the periods, in contrast to previous methods [7]. (3) Constrained Fourier with one and two harmonics sufficiently estimated noisy data (similarly to [17]), and (4) we do not recommend to replace Fourier approximation with spline when the periods are unknown (suggested by [7]), because this and similar methods do not use a-priori information of the gene expression and are likely to over-fit noisy data.

Most importantly, our results imply that analysis by network component analysis (NCA) and k-means clustering of untreated, noisy data do not produce reliable predictions. Similar results were shown previously for PCA [38]. Our Monte Carlo simulations indicated that: (1) The NCA could predict consistently from replicates of de-noised data. (2) The information of the original signals is better preserved when de-noising individual noisy signals with constrained Fourier before clustering. This was consistent as the noise variance increased (Fig. 3). k-means clustering of expression signals with high noise variance formed often different cluster shapes than the original (see examples in Additional file 1: Fig. S4). This implies that smoothing the gene expression signals using cross correlation information from clusters generated by highly noisy data (suggested by [7, 16, 17]) may produce inaccurate approximation. At low variance however, our analysis did not reveal any significant difference between clustering individually de-noised genes and clustering noisy genes. Based on these results, we suggest applying Fourier de-noising of individual genes prior to clustering algorithms (for instance k-means) for noisy genes. In contrast, at low noise values the information from k-means clusters of raw data can be valuable to our constrained Fourier and can be used to re-evaluate the estimated functions of individual genes post clustering.

We showed that our Fourier approximation is sensitive to the sampling frequency. Because gene expression measurements demand resources, there is often a trade-off between exploring temporal behavior (many time points) and improving the accuracy at each time point (many replicates and sequencing depth, [12]). Here we showed that fewer than 7 time samples (particularly uniformly distributed sampling frequencies) generated poor Fourier approximations (Fig. 2E, F). Because logarithmic time scale is a common practice in time-series gene expression measurements [28], we suggest to measure at least 5–7 time samples to improve the clustering performance. Importantly, there was little gain in noise reduction efficiency beyond 8 time samples, suggesting we can optimize our resources elsewhere (e.g. more replicates).

The limitations of real data analysis stem mostly from the unknown noise model, which is often difficult to predict. Unlike synthetic data, real measurements often contain colored noise that emerges among other things from correlations between sample acquisition, biased during the sample preparation, and most importantly the effect of time on the samples and the transcriptome. Stochastic fluctuations in gene expression are often assumed to be Gaussian white noise in nature but the zero correlation time for white noise assumes an infinite relaxation time [48]. For instance, following environmental cues on the cells (e.g. heat shock or pressure shock), the gene expression pattern responding to the stimuli with time is diluted by many factors, such as deteriorating state of the cells and their membrane [45], cell differentiation, cellular metabolism and other functions that are not directly measured but are affected by time [49, 50]. To decrease the effect of colored noise, it is recommended to have a large set of control samples to account for this variability. Another option is to measure an increasing level of the same stimuli in an attempt to capture its dynamic effect, and even model the effect of finite correlation time of noise into the study of stochastic fluctuations [48].

Lastly, an extension of the algorithm (under development) clusters the genes using functional PCA [51], and re-estimate individual genes that increase the Rand index error of the cluster space, with different Fourier harmonics and initial conditions. It then tests the cluster’s accuracy and iterates to minimize the error.

## Conclusions

The algorithm and results presented here can provide a robust technique to de-noise time-series gene expression data and have the potential to improve gene expression post processing methods such as PCA and clustering. This increases our chance to discover important network features from the large time-series data generated in the last decade.

## Supplementary Information

**Additional file 1:** Supplementary Tables and Figures.

## Data Availability

The RNA-seq data for Listeria monocytogenes is available in the European Nucleotide Archive (ENA) under accession code PRJEB34771 [39], while the mouse T cell data was downloaded from GEO database, array express accession number: GSE13009 [28]. The TF-TG interaction data was downloaded from TFactS database [52] and HTRIdb database [53]. The algorithms of the analysis are available in Matlab at the author’s website.

## Abbreviations

ATF: Activating transcription factor
DEG: Differentially expressed genes
GNCA-r: General NCA
ISNCA: Iterative sub-network component analysis
NCA: Network component analysis
NIPALS: Nonlinear iterative partial least squares
PCA: Principle component analysis
ROBNCA: Robust NCA
TG: Target gene
TF: Transcription factor
