# Supplementary material for: Constrained Fourier estimation of short-term time-series gene expression data reduces noise and improves clustering and gene regulatory network predictions
Source: BMC Bioinformatics. 2022 Aug 9;23:330. doi: 10.1186/s12859-022-04839-z (PMC9364503; doi:10.1186/s12859-022-04839-z)
Supplement: Supplementary file 1 — Additional file 1: Supplementary Tables and Figures. [file 12859_2022_4839_MOESM1_ESM.pdf]

Supplementary Tables

Table S1 Mean SSE values computed between noisy or de-noised signals and real synthetic signals. Standard deviation is shown in brackets.

	All time points			"High noise" time points		
	Noisy data	ImpulseDE	Constrained Fourier	Noisy data	ImpulseDE	Constrained Fourier
Cluster 1	0.36 (±0.15)	0.29 (±0.13)	0.22 (±0.13)	0.35 (±0.15)	0.27 (±0.13)	0.21 (±0.13)
Cluster 2	0.49 (±0.18)	0.34 (±0.15)	0.29 (±0.15)	0.48 (±0.19)	0.33 (±0.15)	0.28 (±0.15)
Cluster 3	0.36 (±0.14)	0.24 (±0.11)	0.19 (±0.11)	0.36 (±0.14)	0.23 (±0.11)	0.17 (±0.11)
Cluster 4	0.34 (±0.14)	1.08 (±0.04)	0.47 (±0.06)	0.33 (±0.14)	0.90 (±0.08)	0.32 (±0.10)
Cluster 5	0.51 (±0.14)	0.33 (±0.11)	0.27 (±0.12)	0.51 (±0.15)	0.31 (±0.11)	0.25 (±0.12)
Cluster 6	0.26 (±0.13)	0.23 (±0.11)	0.55 (±0.05)	0.26 (±0.13)	0.22 (±0.12)	0.41 (±0.09)

Supplementary Figures

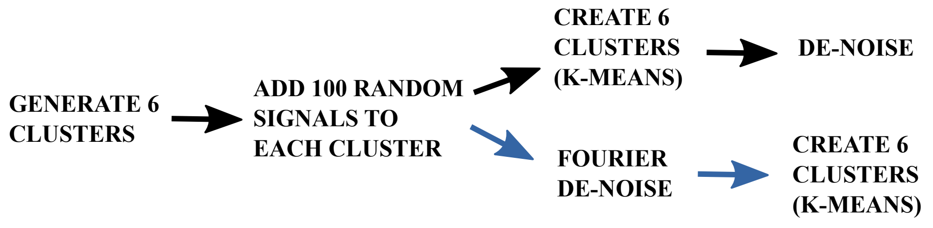

Figure S1 Two de-noising methods: i) De-noising gene expression data by clustering first (top), then using cross-correlation from each cluster to constrain the smoothing algorithm, and ii) de-noising individual genes with algorithm 1, then apply clustering (bottom).

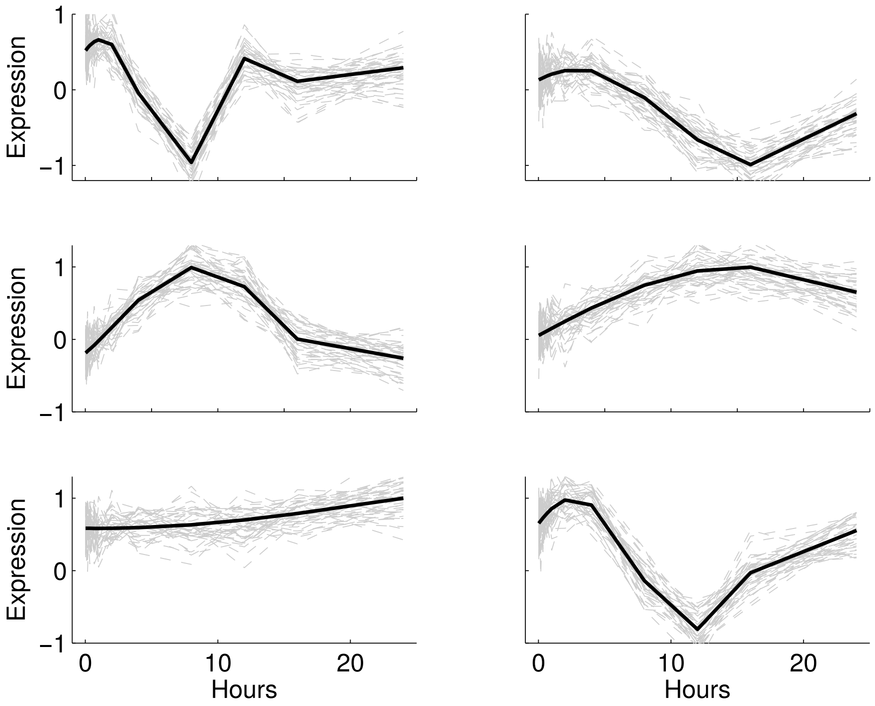

Figure S2 Synthetically generated noisy gene clusters. Six generated, randomly chosen frequencies from uniform distribution with low (< 0.4) cross-correlated matrix values (solid black). The genes that associated with each cluster were generated by random noise (Gaussian) of the above frequencies, according to the noise model (Eq. 1) with variances $\sigma^2 = 0.2$ and $\phi^2 = 0.1$.

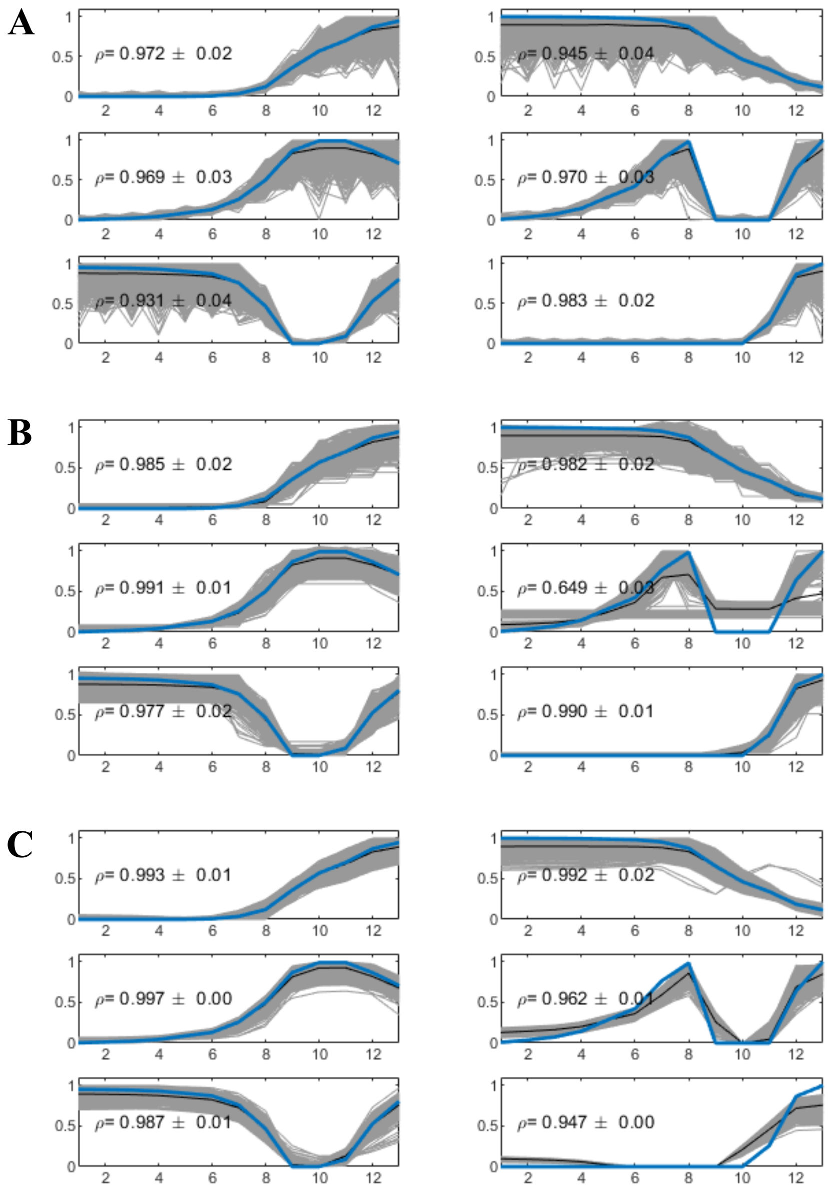

Figure S3 De-noising of synthetic RNA-seq gene expression data. A) 1000 genes were created for each cluster using a negative binomial distribution. B) De-noised gene profiles using ImpulseDE. C) De-noised gene profiles using our constrained Fourier method. Mean correlation coefficients ρ were calculated between raw or de-noised signals and true signal (blue lines). Black lines show the mean calculated over all single genes (raw or de-noised) of one cluster.

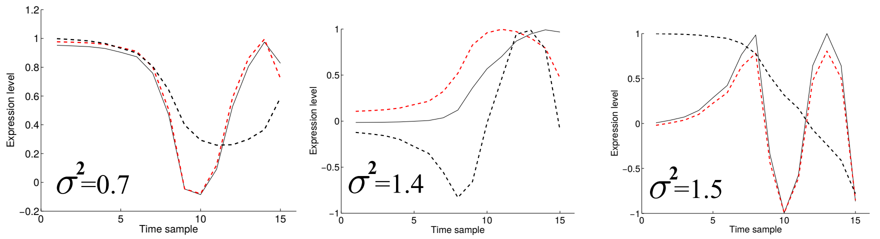

Figure S4 Comparison of Fourier approximated high noisy signals ($\sigma^2 > 0.5$), by clustering raw data (dashed gray) and de-noised data (red dashed) to the original signal (solid).

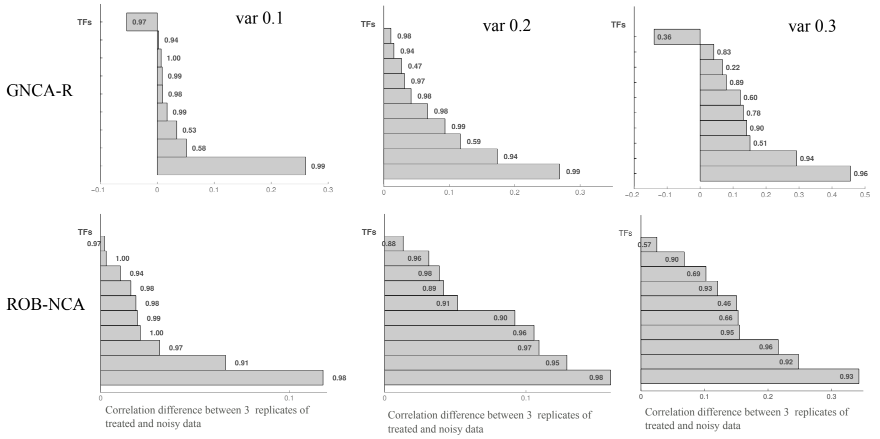

Figure S5 GNCA-r and ROB-NCA network analysis performed better when data was treated with NR for all noise variances we tested. Positive values show improvement of the NR over the non-treated gene expression data. Rows are the NCA algorithm we tested, and columns are the different variances. When reconstructed 10 transcription factor (TF) signals from 3 replicates of data, the correlation between the replicates was higher when the data was first treated with our constrained Fourier estimation. ROB-NCA performed better after noise-reduction. The numbers attached to the columns are the correlation values of 3 replicates from treated data. Note that only 9 TF are presented in the GNCA-r with variance 0.1 due to strong outlier in one of the simulations.
